# The neutrophil-to-lymphocyte ratio is an important indicator correlated to early neurological deterioration in single subcortical infarct patients with diabetes

**DOI:** 10.3389/fneur.2022.940691

**Published:** 2022-10-20

**Authors:** Lijun Fang, Yali Wang, Hong Zhang, Lingling Jiang, Xuehong Jin, Yongquan Gu, Minya Wu, Shaofang Pei, Yongjun Cao

**Affiliations:** ^1^Department of Neurology, The Second Affiliated Hospital of Soochow University, Suzhou, China; ^2^Department of Neurology, Suzhou Municipal Hospital, Nanjing Medical University, Suzhou, China

**Keywords:** single subcortical infarction, early neurological deterioration, neutrophil-to-lymphocyte ratio, diabetes, branch atheromatous disease, cerebral small vessel disease

## Abstract

**Background and purpose:**

This study aimed to investigate the relationship between neutrophil-to-lymphocyte ratio (NLR) and early neurological deterioration (END) among cases suffering from single subcortical infarction (SSI) and diabetes.

**Methods:**

We collected the data of patients with SSI admitted to our hospital between January 2019 and December 2020 retrospectively. A score of ≥2 elevations in overall National Institutes of Health Stroke Scale (NIHSS) score or ≥1 increase in motor NIHSS score in 5-day post-admission was considered END. Furthermore, logistic regression was used to analyze the relationship between NLR and END among SSI cases.

**Results:**

Altogether, we enrolled 235 consecutive SSI cases, of which 53 (22.5%) were diagnosed with END, while 93 (39.5%) were diabetic. In patients with diabetes, the value of NLR increased markedly among the patients with END (median, 3.59; IQR, 2.18–4.84) compared to patients without END (median, 2.64; IQR, 1.89–3.18; *P* = 0.032). Meanwhile, in patients without diabetes, NLR was not significantly associated with END. In the multivariate analysis, NLR values were positively related to END (adjusted odds ratio (OR), 1.768; 95% CI, 1.166–2.682, *P* = 0.007) upon adjusting age, SSI type, lesion diameter, initial NIHSS, fasting blood glucose (FBG), 2-h postprandial blood glucose (2hPBG), and estimated glomerular filtration rate (eGFR). The subgroup analysis showed that the relationship between NLR and END was more pronounced in the branch atheromatous disease (BAD) (adjusted OR, 1.819; 95% CI, 1.049–3.153, *P* = 0.033) and anterior SSI subgroups (adjusted OR, 2.102; 95% CI, 1.095–4.037, *P* = 0.026).

**Conclusion:**

NLR value was significantly related to END among SSI patients with diabetes and was recognized as an independent factor in predicting the risk of END.

## Introduction

A single subcortical infarction (SSI) (1) is an ischemic lesion that is found at the level of a single perforating artery. However, it usually has a favorable prognosis. SSI has two main pathological types, including branch atheromatous disease (BAD) (2), mainly related to atherosclerosis (AS), and cerebral small vessel disease (CSVD) (3), which is mainly related to lipohyalinosis and has a smaller lesion than BAD.

Although the symptoms of SSI are usually mild, the dysfunctions of motor and sensory function are quite common (4). During the acute phase of stroke, approximately 13.5–47.5% of patients with SSI experience worsening neurological deficits (5), especially in motor function. This is known as early neurological deterioration (END), which can aggravate physical disability after stroke and can also prolong the recovery time (6).

Diabetes was identified as an independent factor in predicting stroke. Stroke cases suffering from diabetes have worse motor functions and also poor outcomes (7). Insulin resistance (IR), glucotoxicity, and lipotoxicity may cause systemic chronic low-grade inflammation and can be related to AS and lipohyalinosis (8, 9). After the occurrence of cerebral ischemia, the pro-inflammatory process is further accelerated, leading to the aggravation of ischemic injury (10).

The neutrophil-to-lymphocyte ratio (NLR) serves as the cheap, facile, widely accessible, and highly stable factor reflecting systemic inflammation, which is less susceptible to physiological states, such as hemoconcentration (11). An increased NLR is related to serious stress, inflammation, trauma, injury, cancer, or major surgery, which can also predict a poor prognostic outcome in terms of incidence and mortality (11). NLR is a new indicator of subclinical inflammation in diabetes (12). An increased NLR was significantly associated with IR (13), and its value acted as a predictive marker, indicating the risk and severity of diabetic complications, such as diabetic retinopathy (14), diabetic nephropathy (15), diabetic peripheral neuropathy (16), and coronary microvascular dysfunction (17).

The NLR shows positive relation to 3-month mortality risk post-stroke (18). An increasing number of studies have reported the sensitivity of NLR in predicting stroke severity and short-term prognosis (19–21). However, there are few studies on the role of NLR in cerebrovascular complications of diabetes. Luo et al. confirmed that NLR is an independent risk factor for cerebral hemorrhage in patients with type 2 diabetes (T2DM) (22). However, the role of NLR in ischemic stroke patients with diabetes is rarely investigated.

Hence, this study was focused on determining the potential role of NLR in the END among patients with SSI and diabetes to facilitate early screening and treatment.

## Methods

### Study design and patients

In this retrospective observational study, we enrolled patients with acute ischemic stroke admitted to Suzhou Municipal Hospital between January 2019 and December 2020. The inclusion criteria were as follows: (1) age ≥ 18 years; (2) acute SSI confirmed by diffusion-weighted magnetic resonance imaging (MRI), where the duration between symptom occurrence and admission was less than 72 h; (3) complete cerebral vascular examination (computed tomography angiography (CTA) or MR angiography); and (4) the diameter of the lesion being ≤20 mm. The patients who received thrombolytic and thrombectomy therapy and had cardiogenic cerebral embolism (CCE), severe basilar artery or middle cerebral artery stenosis (over 50% stenosis rate), severe carotid arterial stenosis (over 50% stenosis rate), malignant tumor, severe kidney or liver disease, hematological disease, or inflammatory or infectious disease were excluded from the study. Our study was approved by the Medical Ethics Committee of Suzhou Municipal Hospital.

### Stroke severity and assessment of the END

We assessed the severity of stroke daily using the National Institutes of Health Stroke Scale (NIHSS) score from admission to discharge by two trained neurologists not involved in this study, and disagreements were resolved by discussion with a third neurologist. According to the initial NIHSS score, all patients were classified into END and non-END groups. In this study, END was considered if there was an increase of ≥2 in overall NIHSS score or an increase of ≥1 in motor NIHSS score within 5 days of admission (23, 24). In contrast, the patients with an increase of <2 in overall NIHSS score and no increase in motor NIHSS score within 5 days of admission were assigned to the non-END group.

### Clinical and laboratory data collection

Upon admission, we harvested the baseline data of patients, including demographic data, risk factors for stroke, laboratory results, and clinical information. We identified risk factors, such as diabetes, hypertension, previous stroke history, coronary artery disease, dyslipidemia, and drinking and smoking history. In our study, diabetic patients included those with previous diagnosis of diabetes and the patients of HbA1c ≥6.5% with no history of diabetes (25). In addition, we recorded the laboratory results of fasting blood drawn the next morning after admission, which included the values of white blood cells, hs-CRP, neutrophils (N), lymphocytes (L), glycosylated hemoglobin (HbA1c), fasting blood glucose (FBG), 2-h postprandial blood glucose (2hPBG), triglycerides (TG), total cholesterol (TC), high-/low-density lipoprotein (HDL/LDL), and creatinine. In this study, the Chronic Kidney Disease Epidemiology Collaboration (CKD-EPI) formula was used to calculate the baseline estimated glomerular filtration rate (eGFR). Based on the ratio of neutrophil count/lymphocyte count in the peripheral blood, NLR was determined at admission.

### Image analysis

**Figure 1:**
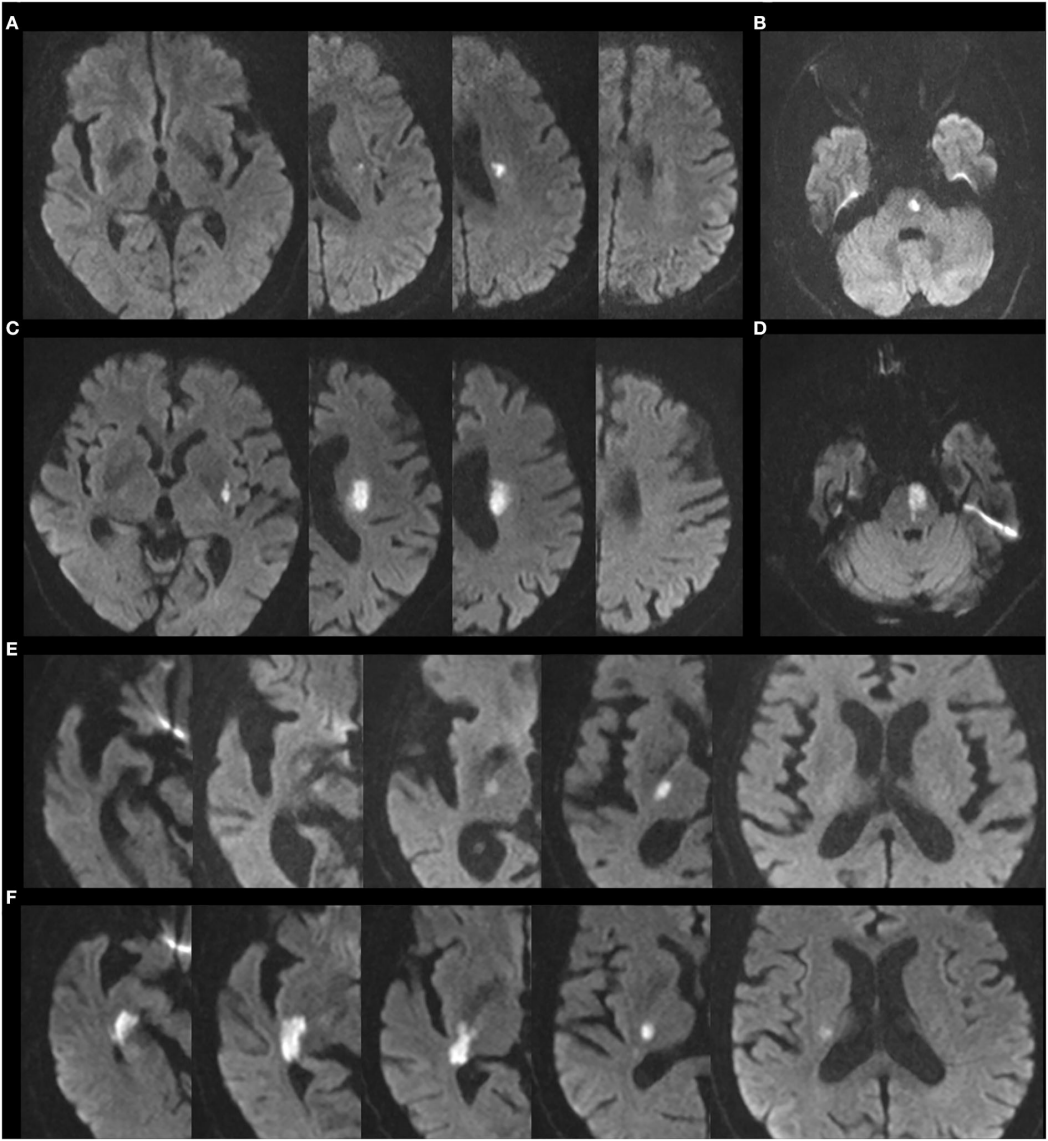
Representative images of BAD and CSVD lesion supplied by LSA, AChA, and PPA. **(A)** CSVD lesion supplied by LSA. **(B)** CSVD lesion supplied by PPA. **(C)** BAD lesion supplied by LSA. **(D)** BAD lesion supplied by PPA. **(E)** CSVD lesion supplied by AChA. **(F)** BAD lesion supplied by AChA.

Each patient underwent brain MRI (AVANTO; Siemens, Germany), and CTA involving the brain and neck using the Intellispace Portal (Philips, the Netherlands) within 48 h after admission. Two experienced neurologists (LF and YW), blinded for clinical data, evaluated the lesion diameter and the lesion depth of infarct lesions on diffusion-weighted imaging, and stenosis of parent artery on CTA. SSI was defined as a single lesion supplied by lenticulostriate artery (LSA), anterior choroidal artery (AChA), and paramedian pontine artery (PPA) in this study (25). According to the characteristics of infarct lesions, patients were divided into BAD and CSVD subgroups (Figure 1). The BAD subgroup (26) mainly included patients meeting the following criteria: lesions supplied by LSA with a diameter of ≥15 mm and ≥3 layers (slice thickness of 5 mm) and without any severe middle cerebral artery stenosis (stenosis rate of >50%); those supplied by AChA with a diameter of ≥15 mm and ≥3 layers (slice thickness of 5 mm) and without any severe internal carotid artery (ICA) stenosis in the distal end (stenosis rate of > 50%); and those supplied by PPAs extending to the surface of the basal pons and without severe stenosis of the basilar artery (stenosis rate of >50%). In contrast, the CSVD subgroup included other patients with lesions supplied by LSA, AChA, or PPA.

### Statistical analysis

SPSS version 26.0 (IBM Corporation) was employed for statistical analysis. Normally distributed quantitative data were presented as mean ± standard deviation (SD). Student's *t*-test was used for comparison. Abnormally distributed data were expressed by the median and interquartile range (IQR), followed by a comparison using the Mann–Whitney *U* Test. A chi-square test was utilized to compare categorical data. Both univariate and multivariate binary logistic regression analyses were used to evaluate the association between NLR and END. The results were represented using odds ratios (ORs) with relevant 95% confidence intervals (CIs). A difference of *P* < 0.05 indicated statistical significance.

## Results

### General characteristics

In this study, we included all 235 consecutive cases suffering from SSI between January 2019 and December 2020. Based on the increase in the NIHSS score, the patients were classified into END and non-END groups, as described previously. Table 1 lists the baseline clinical parameters of enrolled patients, with END being diagnosed in 53 (22.5%) cases. The age and sex of the patient did not exhibit any significant difference between both groups. Similarly, vascular risk factors, including hyperlipidemia, diabetes, and hypertension, also did not exhibit any difference. The distributions of FBG, 2hPBG, HbA1c, TG, TC, HDL, LDL, creatinine, eGFR, white blood cells, hs-CRP, and NLR were similar between both groups. Compared to the non-END cases, patients with END showed an increased initial NIHSS score (*P* < 0.001) along with increased diameter (*P* < 0.001) and length (*P* = 0.005) of the ischemic lesion. Moreover, the BAD group showed a significant increase in the percentage of stroke among patients with END, exhibiting a significant difference.

**Table 1:** Clinical characteristics of general patients in the non-END and END groups.

	**Non-END (*n =* 182)**	**END (*n =* 53)**	***P*-value**
Age (years)	65.96 ± 11.40	68.04 ± 10.64	0.222
Male, *N* (%)	122 (67.0%)	34 (64.2%)	0.696
Onset to END time (days)		4.00 (3.50–5.00)	
Onset to NLR testing time (hours)	50.00 (41.75–72.00)	48.00 (42.00–67.00)	0.484
BMI (kg/m^2^)	23.92 ±3.35	25.14 ±3.31	0.055
Hypertension, *N* (%)	151 (83.0%)	45 (84.9%)	0.738
Diabetes, *N* (%)	71 (39.0%)	22 (41.5%)	0.743
Hyperlipidemia, *N* (%)	52 (28.6%)	9 (17.0%)	0.090
Coronary disease, *N* (%)	16 (8.8%)	1 (1.9%)	0.088
Previous stroke, *N* (%)	39 (21.4%)	11 (20.8%)	0.916
Smoking, *N* (%)	83 (45.6%)	22 (41.5%)	0.598
Drinking, *N* (%)	50 (27.5%)	14 (26.4%)	0.879
Initial NIHSS score	3.00 (2.00–4.00)	4.00 (3.00–5.00)	<0.001
Type of SSI, *N* (%)			<0.001
CSVD	106 (58.2%)	12 (22.6%)	
BAD	76 (41.8%)	41 (77.4%)	
Lesion location, *N* (%)			0.714
LSA	89 (48.9%)	23 (43.4%)	
AChA	38 (20.9%)	11 (20.8%)	
PPA	55 (30.2%)	19 (35.8%)	
Lesion diameter (mm)	12.00 (9.00–17.00)	17.00 (13.00–19.00)	<0.001
Lesion depth (slide n)	2.00 (1.00–3.00)	2.00 (2.00–3.00)	0.005
FBG (mmol/L)	5.44 (4.81–6.93)	6.32 (4.90–8.59)	0.083
2hPBG (mmol/L)	7.80 (6.10–10.60)	7.80 (6.30–12.00)	0.399
HbA1c (%)	5.90 (5.50–7.14)	5.97 (5.50–8.00)	0.729
TC (mmol/L)	4.57 (3.90–5.54)	4.53 (3.92–5.28)	0.569
TG (mmol/L)	1.25 (1.01–1.96)	1.18 (0.94–1.62)	0.262
HDL (mmol/L)	1.03 (0.87–1.21)	1.01 (0.87–1.18)	0.672
LDL (mmol/L)	2.91 (2.31–3.39)	2.71 (2.29–3.36)	0.823
Creatinine (umol/L)	74.40 (60.10–87.15)	70.75 (56.70–80.02)	0.134
eGFR (mL·min^−1^·1.73m^−2^)	88.39 (73.83–98.33)	87.25 (80.28–96.02)	0.589
White blood cells (× 10^9^/L)	6.59 (5.37–7.95)	6.66 (5.32–8.50)	0.694
hs-CRP (mg/L)	1.66 (0.94–3.84)	2.40 (0.91–5.17)	0.377
NLR	2.51 (1.82–3.36)	3.01 (2.03–4.46)	0.098

END, early neurological deterioration; BMI, body mass index; NIHSS, National Institutes of Health Stroke Scale; SSI, single subcortical infarction; CSVD, cerebral small vessel disease; BAD, branch atheromatous disease; LSA, lenticulostriate artery; AChA, anterior choroidal artery; PPA, paramedian pontine arteries; FBG, fasting blood glucose; 2hPBG, 2-h postprandial blood glucose; TC, total cholesterol; TG, triglyceride; HDL, high-density lipoprotein; LDL, low-density lipoprotein; eGFR, estimated glomerular filtration rate; hs-CRP, high-sensitivity C-reactive protein; NLR, neutrophil-to-lymphocyte ratio.

### Baseline characteristics in patients with SSI and diabetes

The factors related to END were explored in SSI patients with diabetes by further comparing the baseline clinical parameters between patients with diabetes (*n* = 93) and without diabetes (*n* = 142) (Table 2). An increase was observed in the NLR value in patients with diabetes (*P* < 0.05) but not in patients without diabetes having END. Table 2 also presents additional baseline features.

**Table 2:** Clinical characteristics of non-END and END groups in SSI patients with and without diabetes.

	**Non-diabetic SSI (*****n** =* **142)**			**diabetic SSI (*****n** =* **93)**		
	**Non-END (*n =* 111)**	**END (*n =* 31)**	***P*-value**	**Non-END (*n =* 71)**	**END (*n =* 22)**	***P*-value**
Age (years)	65.74 ± 12.12	68.71 ± 11.35	0.224	66.31 ± 10.25	67.09 ± 9.73	0.753
Male, *N* (%)	77 (69.4%)	17 (54.8%)	0.131	45 (63.4)	17 (77.3)	0.227
Onset to END time (days)		4.00 (4.00–5.00)			4.00 (3.00–5.25)	
Onset to NLR testing time (hours)	52.00 (42.00–72.00)	48.00 (42.00–72.00)	0.431	48.00 (40.00–72.00)	48.00 (41.75–60.50)	0.924
BMI (kg/m^2^)	23.47 ± 3.31	25.01 ± 3.37	0.065	24.59 ± 3.32	25.31 ± 3.33	0.459
Hypertension, *N* (%)	87 (78.4%)	26 (83.9%)	0.502	64 (90.1%)	19 (86.4%)	0.696
Hyperlipidemia, *N* (%)	24 (21.6%)	5 (16.1%)	0.502	28 (39.4%)	4 (18.2%)	0.077
Coronary disease, *N* (%)	10 (9.0%)	1 (3.2%)	0.287	6 (8.5%)	0 (0%)	0.330
Previous stroke, *N* (%)	24 (21.6%)	4 (12.9%)	0.281	15 (21.1%)	7 (31.8%)	0.303
Smoking, *N* (%)	50 (45.0%)	12 (38.7%)	0.529	33 (46.5%)	10 (45.5%)	0.933
Drinking, *N* (%)	34 (30.6%)	5 (16.1%)	0.110	16 (22.5%)	9 (40.9%)	0.089
Initial NIHSS score	3.00 (2.00–4.00)	4.00 (3.00–5.00)	0.005	2.00 (2.00–5.00)	4.00 (3.00–5.25)	0.023
Type of SSI, *N* (%)			<0.001			0.033
CSVD	65 (58.6%)	5 (16.1%)		41 (57.7%)	7 (31.8%)	
BAD	46 (41.4%)	26 (83.9%)		30 (42.3%)	15 (68.2%)	
Lesion location, *N* (%)			0.462			0.949
LSA	60 (54.1%)	14 (45.2%)		29 (40.8%)	9 (40.9%)	
AChA	27 (24.3%)	7 (22.6%)		11 (15.5%)	4 (18.2%)	
PPA	24 (21.6%)	10 (32.3%)		31 (43.7%)	9 (40.9%)	
Lesion diameter (mm)	12.00 (9.00–16.00)	17.00 (13.00–19.00)	<0.001	11.00 (9.00–17.00)	16.00 (10.75–18.25)	0.031
Lesion depth (slide n)	2.00 (1.00–3.00)	2.00 (2.00–3.00)	0.154	2.00 (1.00–2.00)	2.00 (2.00–3.00)	0.004
FBG (mmol/L)	5.07 (4.67–5.47)	5.26 (4.81–5.74)	0.239	7.29 (6.17–11.19)	8.29 (6.75–10.87)	0.324
2hPBG (mmol/L)	6.25 (5.62–7.67)	6.65 (5.87–7.20)	0.641	11.20 (9.10–14.20)	12.70 (10.15–13.50)	0.319
HbA1c (%)	5.55 (5.26–5.80)	5.50 (5.15–5.89)	0.927	7.43 (6.50–9.31)	8.00 (6.72–9.49)	0.602
TC (mmol/L)	4.58 (3.89–5.31)	4.56 (3.52–5.36)	0.732	4.55 (3.90–5.75)	4.49 (3.94–5.19)	0.668
TG (mmol/L)	1.14 (0.92–1.63)	1.02 (0.85–1.39)	0.333	1.66 (1.10–2.45)	1.43 (1.08–2.14)	0.453
HDL, (mmol/L)	1.04 (0.92–1.26)	1.00 (0.88–1.26)	0.501	1.01 (0.85–1.15)	1.04 (0.86–1.17)	0.740
LDL (mmol/L)	2.86 (2.28–3.36)	2.89 (2.27–3.35)	0.859	2.94 (2.41–3.50)	2.67 (2.35–3.57)	0.552
Creatinine (umol/L)	76.25 (60.90–87.87)	70.50 (55.60–81.30)	0.064	71.20 (57.25–85.82)	71.10 (58.95–77.45)	0.880
eGFR (mL·min^−1^·1.73m^−2^)	86.10 (73.32–97.08)	86.54 (78.34–94.96)	0.695	89.19 (74.60–99.83)	90.61 (82.59–103.24)	0.658
White blood cells (× 109/L)	6.42 (5.00–7.50)	6.44 (5.15–8.07)	0.643	7.00 (5.79–8.18)	7.35 (5.64–8.77)	0.736
Hs-CRP (mg/L)	1.55 (0.80–3.66)	1.79 (0.88–4.63)	0.565	1.75 (0.94–3.92)	3.17 (0.98–6.31)	0.293
NLR	2.37 (1.76–3.79)	2.41 (1.78–4.39)	0.585	2.64 (1.89–3.18)	3.59 (2.18–4.84)	0.032

END, early neurological deterioration; BMI, body mass index; NIHSS, National Institutes of Health Stroke Scale; SSI, single subcortical infarction; CSVD, cerebral small vessel disease; BAD, branch atheromatous disease; LSA, lenticulostriate artery; AChA, anterior choroidal artery; PPA, paramedian pontine arteries; FBG, fasting blood glucose; 2hPBG, 2-h postprandial blood glucose; TC, total cholesterol; TG, triglyceride; HDL, high-density lipoprotein; LDL, low-density lipoprotein; eGFR, estimated glomerular filtration rate; hs-CRP, high-sensitivity C-reactive protein; NLR, neutrophil-to-lymphocyte ratio.

### Association between NLR and END in patients with SSI and diabetes

In patients with SSI and diabetes, 22 (23.6%) patients were diagnosed with END, as mentioned earlier, where the NLR values showed a significant increase among the END cases (median, 3.59; IQR, 2.18–4.84) compared to the non-END cases (median, 2.64; IQR, 1.89–3.18; *P* = 0.032) (Table 2). Univariate logistic regression analysis suggested that END was related to NLR (OR, 1.716; 95% CI, 1.172–2.513, *P* = 0.006), lesion diameter (OR, 1.133; 95% CI, 1.016–1.263, *P* = 0.025), and SSI type (OR, 2.929; 95% CI, 1.063–8.067, *P* = 0.038) (Table 3). According to multivariate logistic regression, NLR was remarkably related to END (adjusted OR (aOR), 1.768; 95% CI, 1.166–2.682, *P* = 0.007) upon adjusting age, SSI type, lesion diameter, initial NIHSS, FBG, 2hPBG, and eGFR (as shown in Table 3). Therefore, we suggested NLR as an independent risk factor in predicting END.

**Table 3:** Logistic regression analysis of the factors associated with END in SSI patients with diabetes.

	**Crude OR (95% CI)**	***P*-value**	**Adjusted OR (95% CI)**	***P*-value**
Age	1.008 (0.961–1.057)	0.750	0.999 (0.937–1.065)	0.964
Initial NIHSS	1.196 (0.965–1.481)	0.102	1.073 (0.809–1.423)	0.626
NLR	1.716 (1.172–2.513)	0.006	1.768 (1.166–2.682)	0.007
BAD	2.929 (1.063–8.067)	0.038	0.577 (0.075–4.410)	0.596
Lesion diameter	1.133 (1.016–1.263)	0.025	1.223 (0.965–1.551)	0.096
FBG	1.023 (0.902–1.161)	0.722	1.024 (0.825–1.272)	0.827
2hPBG	1.046 (0.910–1.203)	0.524	1.036 (0.826–1.298)	0.761
eGFR	1.004 (0.979–1.031)	0.747	0.998 (0.966–1.032)	0.913

NIHSS, national institutes of health stroke scale; NLR, neutrophil-lymphocyte ratio; BAD, branch atheromatous disease; FBG, fasting blood glucose; 2hPBG, 2-hour postprandial blood glucose; eGFR, estimated glomerular filtration rate.

Upon adjusting the multivariate analysis for confounding factors in the SSI type-stratified subgroup analysis, NLR values were significantly related to END in patients with BAD (aOR, 1.819; 95% CI, 1.049–3.153, *P* = 0.033) (as shown in Table 4). Moreover, no statistically significant difference was observed among CSVD cases. In addition, NLR was markedly related to END among cases with anterior circulation (aOR, 2.102; 95% CI, 1.095–4.037, *P* = 0.026) (Table 5), but the difference was not statistically significant in patients with posterior circulation (as shown in Table 5).

**Table 4:** Logistic regression analysis of factors associated with END in the BAD and CSVD subgroups.

	**CSVD**		**BAD**	
	**Adjusted OR (95% CI)**	***P*-value**	**Adjusted OR (95% CI)**	***P*-value**
Age	0.904 (0.784–1.042)	0.165	1.033 (0.950–1.124)	0.445
Initial NIHSS	0.941 (0.484–1.829)	0.858	1.095 (0.756–1.586)	0.631
NLR	1.489 (0.649–3.417)	0.347	1.819 (1.049–3.153)	0.033
Lesion diameter	1.534 (0.990–2.376)	0.055	1.122 (0.799–1.577)	0.506
FBG	1.092 (0.727–1.641)	0.672	0.952 (0.707–1.281)	0.743
2hPBG	1.000 (0.682–1.467)	0.999	1.085 (0.791–1.487)	0.613
eGFR	0.955 (0.900–1.012)	0.121	1.026 (0.972–1.084)	0.352

NIHSS, national institutes of health stroke scale; NLR, neutrophil-lymphocyte ratio; BAD, branch atheromatous disease; FBG, fasting blood glucose; 2hPBG, 2-hour postprandial blood glucose; eGFR, estimated glomerular filtration rate.

**Table 5:** Logistic regression analysis of factors associated with END in the posterior and anterior SSI subgroups.

	**Posterior SSI**		**Anterior SSI**	
	**Adjusted OR (95% CI)**	***P*-value**	**Adjusted OR (95% CI)**	***P*-value**
Age	0.947 (0.840–1.067)	0.369	0.997 (0.909–1.093)	0.944
Initial NIHSS	0.869 (0.513–1.473)	0.602	1.157 (0.766–1.746)	0.489
NLR	2.756 (0.950–7.996)	0.062	2.102 (1.095–4.037)	0.026
BAD	0.190 (0.005–7.198)	0.371	0.792 (0.027–22.954)	0.892
Lesion diameter	1.570 (0.980–2.515)	0.060	1.250 (0.793–1.968)	0.336
FBG	1.076 (0.779–1.486)	0.657	1.120 (0.775–1.620)	0.545
2hPBG	1.062 (0.731–1.543)	0.753	0.894 (0.630–1.269)	0.531
eGFR	1.052 (0.977–1.133)	0.178	0.969 (0.919–1.021)	0.235

NIHSS, national institutes of health stroke scale; NLR, neutrophil-lymphocyte ratio; BAD, branch atheromatous disease; FBG, fasting blood glucose; 2hPBG, 2-hour postprandial blood glucose; eGFR, estimated glomerular filtration rate.

## Discussion

In our study, the NLR value was higher in the END group having SSI and diabetes, while this phenomenon was not seen among patients without diabetes. Moreover, NLR was considered an independent risk factor for END. In addition, the association between NLR and END was found to be more pronounced in the BAD and anterior SSI subgroups.

The neutrophil-to-lymphocyte ratio is considered an indicator of systemic inflammation (11). Recently, NLR was also found to be related to short- and long-term outcomes of ischemic stroke (20, 27). Song et al. reviewed 37 articles in a meta-analysis, which involved 43,979 subjects. The results showed an increase in the baseline NLR value, which was related to a higher ischemic stroke incidence along with poor outcomes at 3 months (28). After the post-stroke reperfusion treatment, a higher NLR was linked to cerebral edema and clinical deterioration in the short run (29). A retrospective cross-sectional study revealed that NLR was related to END in patients with SSI, especially in proximal and anterior SSI (30). In our study, the correlation between NLR and END was observed only in SSI patients with diabetes but not in patients without diabetes. Based on the difference between the two studies, we speculated that diabetes might be an important factor affecting the relationship between NLR and END. Recent studies confirmed that the value of NLR was related to the higher incidence of cerebrovascular diseases in adults with diabetes, which was a strong factor in predicting sequelae in ischemic stroke patients with hyperglycemia (31–33).

Diabetes served as an independent factor in predicting stroke and, ultimately, poor outcomes (7). The association between diabetes and END has been detected in case–control (34) and cohort studies (35). The NLR and neutrophil counts in ischemic stroke patients with hyperglycemia were significantly higher than that in patients with euglycemia (33, 36). According to previous articles, the neutrophil count showed a positive correlation with infarct volume and stroke severity among patients with stroke (20, 37). During the acute phase of ischemic stroke, neutrophils moved to the ischemic region and were distributed in subarachnoid space and penetrating arterioles (38). Increased neutrophils in arterioles affect vascular function, such as the drainage of impaired cerebrospinal fluid (CSF) (38). The accumulation of increased neutrophils induced by diabetes may have a potential impact on increased edema. In addition, animal experiments suggested that hyperglycemia promoted the stroke-mediated infiltration of neutrophils priming the thrombo-inflammatory cascade while exacerbating brain injury (39, 40). Elevated neutrophils during diabetes can increase the release of pro-inflammatory factors, inducing brain edema, blood–brain barrier disturbance, or nerve cell mortality (38, 41).

Furthermore, our results revealed that the NLR value was associated with END in the BAD subgroup but not in the CVSD subgroup. Previous studies have shown that the BAD group had a higher possibility of showing END (2). BAD is characterized by AS, mainly caused by endothelial activation and inflammation (10, 42). In patients with BAD, NLR has not been discussed previously. However, other inflammation parameters such as hsCRP were found to be higher in the BAD group, which was correlated with the progression and prognosis of BAD (43, 44). These studies suggested that mediators of inflammation may be involved in the rapid progression of atheromatous lesions, thereby affecting the progression and prognosis of BAD.

Interestingly, the association between NLR value and END differed according to the vascular territory. According to our study, the NLR value served as an independent factor in predicting the risk of END in anterior SSI but not in posterior SSI. Several other studies have also reported similar results (29, 30, 45). The main cause of this phenomenon is the anatomical correlation between infarct volume and corticospinal tract. NLR is reported to be significantly correlated with infarct volume (28, 45). In anterior SSI, the corticospinal tract is easily injured due to a large infarct (46). The corticospinal tract, which is in the posterior circulation, can travel down the ventral surface of the brain stem, which is less affected by infarct volume (46). The infarcts in posterior circulation can be fatal (47) and may cause severe inflammatory responses interfering with the NLR value. Therefore, NLR exhibits a close association with END only in the cases of anterior SSI.

## Limitations

Certain limitations should be noted in this study. First, this was a single-center retrospective study, which might suffer from selection bias. Second, since the sample size of this study was not large, a larger multicenter study may be required in the future. Third, since the NLR was only examined upon admission, the value might have changed during the hospital stay. Our future research needs to dynamically record the NLR values to better confirm the predictive value. Despite these limitations, we are the first to analyze the relationship between NLR on admission and the END in patients suffering from SSI and diabetes, which may exhibit a certain reference value.

## Conclusion

According to our results, an increased value of NLR shows an independent relationship with END among SSI patients with diabetes. Thus, NLR has the advantage of being a novel, simple, and readily available risk factor for END among diabetic and SSI cases.

## Data availability statement

The raw data supporting the conclusions of this article will be made available by the authors, without undue reservation.

## Ethics statement

The studies involving human participants were reviewed and approved by Medical Ethics Committee of Suzhou Municipal Hospital, Nanjing Medical University, Suzhou, China. Written informed consent for participation was not required for this study in accordance with the national legislation and the institutional requirements.

## Author contributions

YC, LF, and YW conceived and designed the study. LF analyzed the data and drafted the manuscript. YW analyzed the data and revised the manuscript. HZ, LJ, XJ, YG, MW, and SP collected the data. All authors approved the final manuscript.

## Funding

This study was supported by grants from the National Natural Science Foundation of China (82171296) and the Discipline Construction Program of the Second Affiliated Hospital of Soochow University (XKTJ-TD202004).

## Conflict of interest

The authors declare that the research was conducted in the absence of any commercial or financial relationships that could be construed as a potential conflict of interest.

## Publisher's note

All claims expressed in this article are solely those of the authors and do not necessarily represent those of their affiliated organizations, or those of the publisher, the editors and the reviewers. Any product that may be evaluated in this article, or claim that may be made by its manufacturer, is not guaranteed or endorsed by the publisher.
